# Supplementary figures and images for: Positive peritoneal lavage fluid cytology based on isolation by size of epithelial tumor cells indicates a high risk of peritoneal metastasis
Source: PeerJ. 2024 Jun 28;12:e17602. doi: 10.7717/peerj.17602 (PMC11216200; doi:10.7717/peerj.17602)

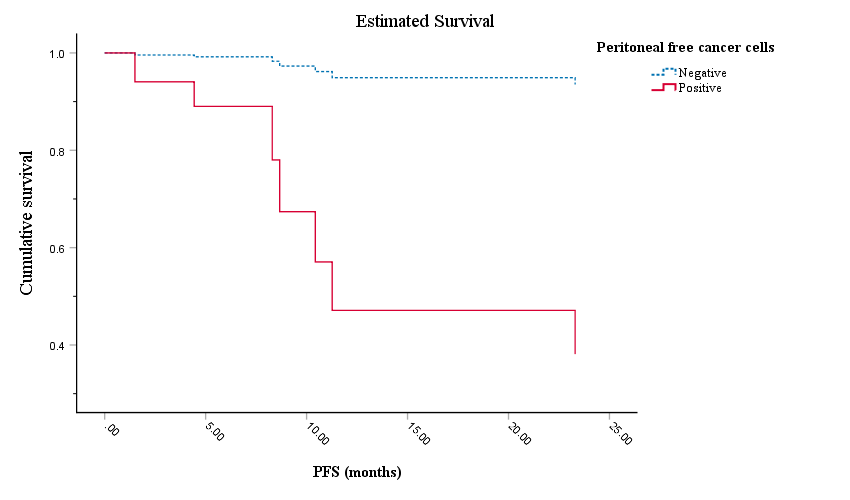

Supplement: Supplemental Information 1 — The Cox regression analysis included potential confounding factors as independent variables, and by adjusting for covariates, significant differences (P=0.023, HR=14.399) exist between the FCCs-positive and negative groups. PFS, progression free survival; FCCs, free cancer cells; GC, gastric cancer; HR, hazard ratio [file peerj-12-17602-s001.png]

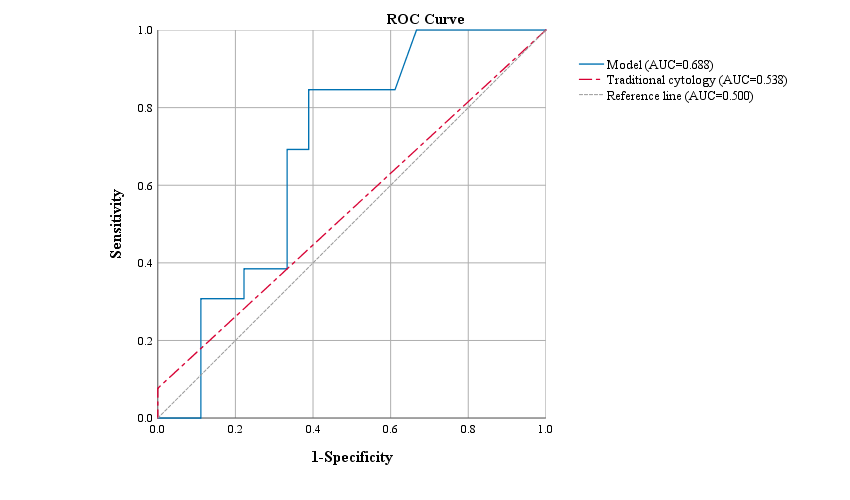

Supplement: Supplemental Information 2 — To predict the patients with recurrence/metastasis, ROC curve was plotted to determine sensitivity and specificity of the model in this study and traditional cytology. ROC, receiver operating characteristic; AUC, area under the curve [file peerj-12-17602-s002.png]
