# Supplementary material for: Positive peritoneal lavage fluid cytology based on isolation by size of epithelial tumor cells indicates a high risk of peritoneal metastasis
Source: PeerJ. 2024 Jun 28;12:e17602. doi: 10.7717/peerj.17602 (PMC11216200; doi:10.7717/peerj.17602)
Supplement: Supplemental Information 3 [file peerj-12-17602-s003.docx]

**Supplement Table 1 Survival of gastric cancer patients in each stage**

| **pTNM stage** | **Total** | **Recurrence and metastasis** | **median PFS (months)** | **Range of PFS (months)** |
| --- | --- | --- | --- | --- |
| Ⅰ-Ⅱ | 16 | 0 | 31.22 | 29.97-34.00 |
| ⅢA-ⅢB | 22 | 6 | 31.45 | 8.30-36.83 |
| ⅢC-Ⅳ | 11 | 7 | 11.27 | 1.50-31.37 |
