# Supplementary material for: Positive peritoneal lavage fluid cytology based on isolation by size of epithelial tumor cells indicates a high risk of peritoneal metastasis
Source: PeerJ. 2024 Jun 28;12:e17602. doi: 10.7717/peerj.17602 (PMC11216200; doi:10.7717/peerj.17602)
Supplement: Supplemental Information 4 [file peerj-12-17602-s004.docx]

| **Supplement Table 2 Univariate analysis of recurrence and metastasis of gastric cancer** | | | | |
| --- | --- | --- | --- | --- |
| **Characteristics** | **Recurrence and metastasis** | | **Univariate analysis** | |
|  | **No** | **Yes** | **χ²** | ***P* value** |
| **Age (y)** |  |  |  |  |
| ＜68 | 16 | 6 | 0.011 | 0.915 |
| ≥68 | 20 | 7 |  |  |
| **Sex** |  |  |  |  |
| Male | 26 | 12 | 1.210 | 0.271 |
| Female | 10 | 1 |  |  |
| **Depth of invasion** |  |  |  |  |
| T1 | 6 | 0 | 4.215 | 0.127 |
| T3 | 6 | 2 |  |  |
| T4 | 24 | 11 |  |  |
| **Lymphatic invasion** |  |  |  |  |
| N0 | 18 | 0 | 16.437 | ＜0.001 |
| N1 | 10 | 4 |  |  |
| N2-3 | 8 | 9 |  |  |
| **pTNM stage** |  |  |  |  |
| Ⅰ-Ⅱ | 18 | 0 | 17.841 | ＜0.001 |
| ⅢA-ⅢB | 14 | 6 |  |  |
| ⅢC-Ⅳ | 4 | 7 |  |  |
| **Venous invasion** |  |  |  |  |
| No | 20 | 0 | 12.203 | ＜0.001 |
| Yes | 16 | 13 |  |  |
| **Nerve invasion** |  |  |  |  |
| No | 20 | 2 | 6.230 | 0.013 |
| Yes | 16 | 11 |  |  |
| **Peritoneal free cancer cells** | |  |  |  |
| Negative | 28 | 10 | 0.000 | 1.000 |
| Positive | 8 | 3 |  |  |
