# Supplementary material for: Positive peritoneal lavage fluid cytology based on isolation by size of epithelial tumor cells indicates a high risk of peritoneal metastasis
Source: PeerJ. 2024 Jun 28;12:e17602. doi: 10.7717/peerj.17602 (PMC11216200; doi:10.7717/peerj.17602)
Supplement: Supplemental Information 5 [file peerj-12-17602-s005.docx]

| **Stage IV cases** | **Differentiation** | **Histological subtype** | **Depth of infiltration** | **Therapy** | **The type of surgery** | **Recurrence and metastasis** |
| --- | --- | --- | --- | --- | --- | --- |
| 1 | poorly differentiated | Mixed adeneuroendocrine carcinoma | full-thickness of the gastric wall | perioperative therapy | R0 resection | peritoneal metastasis and Retroperitoneal lymph node enlargement |
| 2 | poorly differentiated | Tubular adenocarcinoma | from full-thickness of the gastric wall into the fibrofatty tissue outside the serosa | perioperative therapy | R0 resection | peritoneal metastasis |

**Supplement Table 3 The information of stage IV cases**
