# Supplementary material for: Positive peritoneal lavage fluid cytology based on isolation by size of epithelial tumor cells indicates a high risk of peritoneal metastasis
Source: PeerJ. 2024 Jun 28;12:e17602. doi: 10.7717/peerj.17602 (PMC11216200; doi:10.7717/peerj.17602)
Supplement: Supplemental Information 6 — The p value, HR, 95% CI of the one-year, two-year and three-year model were presented in Table S4A-C. We observed discrepancies between the one-year model and the three-year model while the parameters of the multivariable regression analysis at two years remained consistent with the three-year model. [file peerj-12-17602-s006.docx]

**Supplement Table 4 Sensitivity analysis of the one-year and two-year models**

**Supplement Table 4-A**

| **Characteristics** | **P value**  **(one-year)** | **P value**  **(two-year)** | **P value**  **(three-year)** |
| --- | --- | --- | --- |
| Age(y) | 0.807 | 0.045 | 0.045 |
| Sex | 0.905 | 0.514 | 0.514 |
| Lymphatic invasion | 0.405 | 0.787 | 0.787 |
| pTNM stage | 0.812 | 0.020 | 0.020 |
| Nerve invasion | 0.820 | 0.017 | 0.017 |
| Peritoneal free cancer cells | 0.838 | 0.023 | 0.023 |

**Supplement Table 4-B**

|  | **HR**  **(one-year)** | **HR**  **(two-year)** | **HR**  **(three-year)** |
| --- | --- | --- | --- |
| Age(y) | 0.000 | 0.068 | 0.068 |
| Sex | 0.000 | 0.386 | 0.386 |
| Lymphatic invasion | 2.307 | 0.765 | 0.765 |
| pTNM stage | 6.115E+13 | 28.324 | 28.324 |
| Nerve invasion | 1.566E+13 | 14.161 | 14.161 |
| Peritoneal free cancer cells | 1.200E+09 | 14.399 | 14.399 |

**Supplement Table 4-C**

|  | **95% CI**  **(one-year)** | **95% CI**  **(two-year)** | **95% CI**  **(three-year)** |
| --- | --- | --- | --- |
| Age(y) | 0-3.59E+99 | 0.005-0.947 | 0.005-0.947 |
| Sex | 0-8.27E+56 | 0.022-6.730 | 0.022-6.730 |
| Lymphatic invasion | 0.322-16.534 | 0.109-5.374 | 0.109-5.374 |
| pTNM stage | 0-3.48E+127 | 1.675-478.853 | 1.675-478.853 |
| Nerve invasion | 0-8.86E+126 | 1.600-125.367 | 1.600-125.367 |
| Peritoneal free cancer cells | 0-1.14E+96 | 1.445-143.522 | 1.445-143.522 |

The p value, HR, 95% CI of the one-year, two-year and three-year model were presented in Table S4A-C. We observed discrepancies between the one-year model and the three-year model, while the parameters of the multivariable regression analysis at two years remained consistent with the three-year model.
